# A new method for evaluating the effectiveness of micro-mobility protective equipment

**DOI:** 10.12688/openreseurope.20804.1

**Published:** 2025-08-26

**Authors:** Adel Almohammad, Panagiotis Georgakis, Suresh Renukappa

**Affiliations:** 1 School of Architecture and Built Environment, University of Wolverhampton Faculty of Science and Engineering, Wolverhampton, England, WV10 0JP, UK

**Keywords:** micromobility, safety, vulnerable road users – VRUs, protective equipment – PE, effectiveness evaluation, sensor, hazard

## Abstract

Micromobility is one of the most important modes of transportation in urban environments and is widely used in modern cities. It refers to lightweight personal vehicles with a maximum speed of 45 km/h and a maximum weight of 350 kg. Effectively, micromobility safety represents one of the most critical concerns of vulnerable road users (VRUs) such as cyclists and employees of courier companies. Various measures and micromobility protective equipment (PE) have been used to enhance VRU safety and reduce traffic accidents involving VRUs. Therefore, evaluating the effectiveness of PE is very important to ensure that it can successfully prevent or reduce the risk of accidents and injuries. In this paper, we present a novel approach for evaluating the effectiveness of micromobility PE. The proposed data-driven method relies on data collected directly from micro-vehicles and micromobility users using various techniques, including web-based questionnaires, micro-vehicle sensor kits, and micromobility hazard detectors. However, synthetic data were generated and used to demonstrate the practicality of the proposed method. Additionally, an example for presenting the results of measuring the correlations and relationships among the collected data has been introduced and demonstrated so that micromobility users and other stakeholders can understand the effectiveness of each type of PE.

## 1.  Introduction

Micromobility is a transport mode that refers to small, low-speed, lightweight personal vehicles. This may include both human- and electric-powered vehicles such as bicycles and e-scooters. Essentially, users of transport modes such as cyclists and e-scooter riders are less protected in crash and accident situations. Like all other vulnerable road users (VRUs), such as pedestrians and motorcyclists, micromobility users require extra care from other road users (e.g., drivers). The risks of crashes or injuries for cyclists are significantly higher than for drivers; therefore, cycling is considered an unsafe mode of travel ( Lawson *et al.*, 2013). Additionally, between 2008 and 2019, an average of 2200 fatalities occurred among bike riders in the European Union (EU) ( Deck *et al.*, 2019). Therefore, various measures and protective equipment (PE), including helmets, high-visibility clothing, and lights, can be used to improve the safety of micromobility users ( Høye *et al.*, 2020). However, PE should provide protection from injury and facilitate ease of movement, without causing fatigue or discomfort ( de Rome, 2006). 

The reason for crashes between cyclists and vehicles is the failure of drivers to detect cyclists in time to avoid accidents ( Costa *et al.*, 2017). The poor visibility (or conspicuity) of VRUs to other drivers is one of the main reasons for accidents involving VRUs ( Rogé *et al.*, 2019). Therefore, motorcycles can increase visibility to other road users by keeping their lights on during the day. Additionally, the probability of collision between cyclists and motorized vehicles can be reduced if cyclists wear high visibility (e.g., light or white colored) clothing in daylight conditions. Therefore, using visibility aids, such as reflective vests and lights, may improve the detection and recognition distance and the ability of drivers to detect pedestrians and cyclists ( Costa *et al.*, 2017). In addition, helmet users are more likely to use accident prevention equipment, such as lights and high-visibility clothing, under poor visibility conditions ( Hollingworth *et al.*, 2015).

Traumatic brain injury (TBI) is the main cause of mortality after bicycle-related injuries, and it represents three-quarters of deaths and two-thirds of hospital visits in the US ( Joseph *et al.*, 2017). Effectively, there has been an ongoing debate regarding how to prevent head injuries of cyclists in collisions (i.e., with motor vehicles) and whether cycling helmets are efficient in preventing head injuries or reducing the severity of experienced injury ( Bambach *et al.*, 2013). It has been found that using helmets by motorcyclists may reduce head and neck severe injuries by 69% and deaths by 42% ( Morgado *et al.*, 2017). It has been also reported that the effectiveness of helmets are 48%–71% in preventing head injuries ( Axelsson & Stigson, 2019). Therefore, the interactions between cyclists, vehicles, and the environment in the three phases of an accident (pre-event, event, and post-event) can be investigated to develop effective approaches to reduce injuries to cyclists. Principally, bicycle helmets reduce the risk of significant head injury and death in the “event” of a cyclist collision. On the other hand, “pre-event” equipment such as bicycle lights and high-visibility (reflective or fluorescent) clothing may reduce the risk of collision in conditions of reduced visibility ( McGuire & Smith, 2000).

The percentage of adult cyclists who are using helmets in Norway has increased from 32% in 2006 to 56% in 2015 ( Høye *et al.*, 2020). Additionally, many governments have introduced legislation regarding the mandatory use of PE, such as helmets on public roads, to reduce micromobility-related injuries and mortality ( Bambach *et al.*, 2013; Joseph *et al.*, 2017). For instance, in France, helmets have been mandatory for 40 years and gloves have been mandatory since 2016 for motorized 2-wheeler riders ( Wu *et al.*, 2019). However, in Sweden, bicycle helmets have been mandatory since 2005 for children under the age of 15 years ( Axelsson & Stigson, 2019). Therefore, PE aims to enhance VRU safety, promote sustainable micromobility, and reduce traffic accidents involving VRUs. Additionally, evaluating the effectiveness of micromobility PE is very important because it provides the necessary insight to determine whether the PE is achieving its intended purpose.

Section two of this paper discusses and critically analyzes previous studies, highlighting gaps in the research topic. The proposed approach for evaluating the effectiveness of micromobility PE is described and detailed in section three including data collection methods, data processing, and analysis techniques used in this research. A discussion on the implementation of this approach and the presentation of the results are provided in section four. Finally, the conclusions are presented in section five.

## 2.  Background and related work

Several studies have been conducted to investigate the impact of micromobility PE on accidents and injuries. This includes the effectiveness evaluation of some micromobility PE, such as helmets and high-visibility clothing. One study investigated the differences between cyclists who wear helmets and those who do not and evaluated injury prevention measures adopted by cyclists in Oxford, United Kingdom ( McGuire & Smith, 2000). This study was based on an observational survey of cyclists passing a pre-identified point and recorded four measures of injury prevention: use of front or rear lights, high-visibility clothing, and bicycle helmets. Only 3.6% of cyclists were observed to use all four measures, while 34.9% of cyclists used none of them. It was also found that collision prevention measures are more likely to be used by bicycle helmet users under reduced lighting conditions. The relationship between safety measures and pediatric polytrauma severity in road traffic injury was analyzed through a retrospective observational study ( Morgado *et al.*, 2017). This study used hospital data on polytraumatized pediatric patients who were hospitalized after road traffic accidents. It has been found that head injury associated with death in two-wheel vehicle riders is correlated with the absence of PE, such as helmets. In another study, an online questionnaire was used to collect information about cyclists’ cycling behavior and cycling accident-related injuries ( Hollingworth *et al.*, 2015). It has been found that accidents are associated with the use of various safety equipment, and helmet use is related to lower chances of being required for an overnight stay in the hospital. In another study, regression analysis of traumatic brain injury (TBI) data from bicycle-related accidents was employed to evaluate the association of helmet use with the severity of TBI and facial fractures ( Joseph *et al.*, 2017). Helmets have been found to reduce the incidence of TBIs in bicycle-related accidents because bicycle helmets protect cyclists from severe head injuries and can save their lives.

The effectiveness of bicycle helmets in preventing head injuries among cyclists involved in crashes has been examined, and the impact of risky cycling behaviors among helmeted and unhelmeted cyclists has been assessed ( Bambach *et al.*, 2013). This study analyzed relevant police reports on road crashes, hospital admissions, and mortality data. It has been found that the chances of sustaining a head injury increased by 1.98–3.89 times for cyclists who were not wearing a helmet, while helmet use was associated with a reduced risk of head injury by up to 74%. In another study, an advanced methodology was proposed for evaluating bicycle helmets by considering the risk of tissue-level brain injury, realistic impact conditions, and head kinematic parameters ( Deck *et al.*, 2019). This study provides a means of comparing the protective capabilities of helmets using a dedicated rating system. In another study, the impact of using helmets by children bicyclists on injury and accident characteristics was evaluated using emergency care center data regarding children admitted due to bicycle crash injuries ( Axelsson & Stigson, 2019). It has been found that the effectiveness of bicycle helmets was 61% in reducing all head injuries and 45% in reducing facial injuries.

The relationship between crash involvement and cyclists’ safety-equipment use was also investigated ( Høye *et al.*, 2020). It focused on the road behaviors of cyclists and attempted to explore scenarios such as using safety equipment (i.e., bicycle lights, high-visibility clothing, and helmets) that lead to risky (and reckless) or safer behaviors. The surveys showed that the regular use of safety equipment is negatively related to certain types of high-risk behaviors (e.g., listening to music while cycling) and collision involvement. In most bicycle–car collisions, drivers fail to detect cyclists in time to avoid collisions because of the poor visibility of cyclists. Therefore, a study based on a simulated car driving task was conducted to investigate whether wearing a yellow jacket by cyclists enhances the visibility of motorists during daylight hours ( Rogé *et al.*, 2019). Motorists performed a VRU (i.e., cyclist and pedestrian) detection task in a car-driving simulator. Motorists can detect cyclists wearing high-visibility jackets at a greater distance. In another study, the impact of reflective tape application to a bicycle (i.e., to the rear frame and pedal cranks) on bicyclist conspicuity enhancement was evaluated ( Costa *et al.*, 2017). This was done by comparing the bicyclist detection distance in four conditions (control, rear red reflector, high-visibility jacket, and reflective tape). As a result, it was concluded that bicyclist conspicuity and safety at night can be significantly improved by applying reflective tape to the rear bicycle frame.

A retrospective observational study using injury and postal survey data was conducted to assess the effectiveness of motorcyclist protective clothing, including trousers, jackets, knee-high or ankle boots, back protection, and gloves ( Wu *et al.*, 2019). As a result of using Poisson regression to estimate the impact of these protective clothing on injury risk, it has been concluded that these PE can protect users from injuries such as lacerations and abrasions, but not from serious injuries such as fractures, sprains, or dislocations. In another study, a prospective cohort study using an online questionnaire was conducted to quantify bikers’ attitudes towards various factors that might contribute to their choice to use PE ( Pratt *et al.*, 2019). This study analyzed the correlations between these factors and the actual use of PE to assess their relative importance. Results indicated that more than 50% of bikers experienced an injury that required at least one week of work, and the injury severity was well correlated with choice of PE use. Another prospective cohort study was conducted to understand the impact of wearing protective clothing during crashes on the subsequent health conditions and quality of life of motorcyclists ( de Rome *et al.*, 2012). Medical records, interview data, and mailed survey data were used for this study. As a result, there were substantial relationships between post-crash health, well-being, and the use of protective clothing. Motorcyclists wearing jackets (and pants) spent less time in the hospital and had less pain immediately post-crash than those who did not wear the PE. In another study, a cross-sectional analytic study was conducted to quantify the association between the usage of motorcycle clothing and injury in crashes ( De Rome *et al.*, 2011). Participants were recruited through motorcycle repair services and hospitals and data were collected through interviews. As a result, protective clothing used by motorcyclists was related to a reduced risk (and severity) of injury after a crash. Motorcyclists wearing PE (jackets, pants, or gloves) were considerably less likely to be admitted to the hospital after a crash. Moreover, the risk of injury to the upper body, hands, wrists, legs, feet, and ankles was substantially reduced when a body armor was used.

Most studies related to micromobility safety and PE efficiency address the benefits and advantages of these PE in preventing accidents and reducing crash injuries. Nevertheless, these investigations are limited and focus on specific types of PE, such as helmets and high-visibility clothing. Typically, retrospective and prospective observational studies, surveys, and interviews are used as a methodology in these studies. Police traffic accident reports and data, hospital admission data, medical records, injury data, and questionnaire data were used as input data.

This study proposes a new method to assess the effectiveness of micromobility PE and investigate how these PE can improve micromobility safety. Therefore, the outcomes of evaluating the effectiveness of such PE may play a considerable role in reducing traffic accidents involving VRUs, enhancing VRU safety, and promoting sustainable micromobility. This study evaluates the effectiveness of 1) micromobility PE used to reduce the chance of a micro-vehicle crash (or to prevent accidents) and 2) micromobility PE used to reduce the chance of personal injury (or to prevent injuries as a result of accidents). Therefore, this evaluation will give micromobility users insight into the effectiveness of PE and will encourage them to wear and use PE constantly while travelling. In this study, PE that can be used to prevent or reduce the risk of accidents or crash involvement is called PE-preventive (e.g., reflective accessories). However, PE, which can be used to prevent or reduce the effects and risks of injuries as a result of accidents, is called PE-protective (e.g., helmet).

## 3.  Proposed method

Evaluating the effectiveness of micromobility PE can significantly enhance the safety of micromobility and VRUs. Therefore, the effectiveness of different categories and types of PE, including PE-preventive and PE-protective, can be analyzed and estimated. Assessing the effectiveness of PE will provide VRUs with a comprehensive understanding of the merits of each PE category, and will help users select and use the most appropriate PE. Therefore, this study introduces a novel methodology for assessing the effectiveness of various types of micromobility PE according to the road and traffic conditions, features of the PE, and characteristics of VRUs. Effectively, the proposed data-driven approach correlates metrics related to the surroundings of micro-vehicles with the characteristics and features of PE used by micromobility users. This approach employs several procedures and techniques of data collection and analysis to assess the effectiveness of various types of PE.

**Figure 1.**
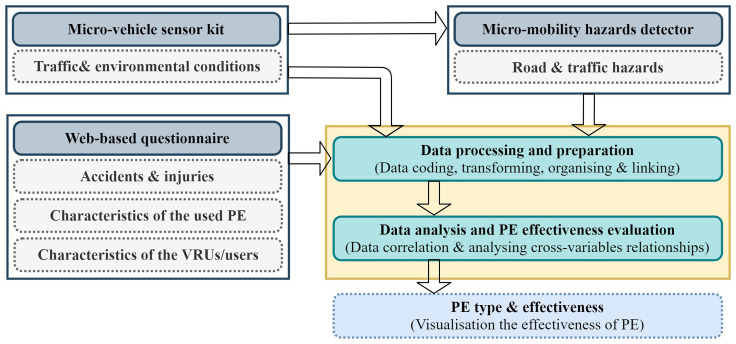
The proposed approach for evaluating the effectiveness of micromobility protective equipment

As mentioned above, most of the previously proposed approaches use historical medical and injury data, hospital admission records, and police traffic accident reports to evaluate the PE efficiency. However, the proposed method is more advanced and relies on near real-time data collected directly from micro-vehicles and users to immediately evaluate the effectiveness of the micromobility PE after finalizing the data collection process. Figure 1 shows the flow diagram for this approach, which depicts the main procedures and steps starting from the data collection process and ending with the effectiveness evaluation of the micromobility protective equipment.

### 3.1  Data collection

This study introduces a data-driven approach for evaluating the effectiveness of various types of micromobility PE. This approach relies mainly on collecting different types of data from several sources. Two main techniques were used to collect the required data. The first technique involves the use of a micro-vehicle sensor kit that can be mounted on a micro-vehicle to collect micro-level data on micromobility, user behavior, and environmental and traffic conditions. However, the second technique involves the deployment of a web-based survey (i.e., questionnaire) to collect data from micromobility users about their behavior on the road, the PE they are using, and the characteristics of these PE.

***3.1.1  Micro-vehicle sensor kit and micromobility hazard detector data***

Various types of sensing systems can be mounted on micro-vehicles to collect safety-related data, including experienced vibration events, proximity to surrounding objects, and speed changes ( Ma *et al.*, 2021). In addition, these systems monitor the status of micro-vehicles, identify riders’ behavior, and detect riding styles ( Han *et al.*, 2022). The proposed method utilizes a micro-vehicle sensor kit and data-driven approach to detect hazards ( Almohammad *et al.*, 2025a). Therefore, this micro-vehicle sensor kit and micromobility hazard detector will be used to collect the first set of data required to evaluate the effectiveness of the micromobility PE. The micro-vehicle sensor kit integrates various devices and sensors such as accelerometers, gyroscopes, GPS receivers, grip sensors, environmental sensors, and depth cameras. However, the micromobility hazard detector uses the data collected by the micro-vehicle sensor kit to detect hazardous situations such as dangerous overtakes. It also identifies areas with specific infrastructure deficiencies (i.e., potholes) and a large number of vehicle conflicts (i.e., heavy traffic). However, other dangerous traffic situations, such as near misses experienced by micromobility users, can be detected by investigating and analyzing camera data and footage.

Accordingly, the output of the micromobility hazard detector is used as input data for the proposed approach to evaluate the effectiveness of PE. This input data includes the number of dangerous overtakes (and may be the severity of these incidents) experienced by micromobility users. Moreover, the proposed approach may use the output of the micro-vehicle sensor kit as input data, including camera footage data, and environmental and weather conditions.

It is expected that many micromobility users will be recruited to participate in the data collection process for the proposed approach. Therefore, many bicycles will be equipped with micro-vehicle sensor kits and used by the recruited users. However, no restrictions are applied on the routes to be followed or the travel time. Consequently, data collected by the micro-vehicle sensor kit will be uploaded to the cloud in real time; therefore, the micromobility hazard detector will be able to read this data and detect related hazards. Principally, data collected by the micro-vehicle sensor kit and the output of the micromobility hazard detector are linked to the responses of micromobility users to the web-based questionnaire.

***3.1.2  Web-based questionnaire data***

The second set of data required for the proposed approach was collected by capturing the responses of micromobility users to a web-based questionnaire. This questionnaire was designed and developed to collect various types of data from micromobility users. As mentioned before, the responses of micromobility users to this questionnaire should be linked to the data provided by the micro-vehicle sensor kit and micromobility hazard detector. Therefore, it is important to use a common data variable that can facilitate the process of linking these datasets together (the response of micromobility users to the questionnaire and the data provided by the micro-vehicle sensor kit and micromobility hazard detector). Micromobility users will be asked to answer ten multiple-choice questions in this questionnaire. Users’ answers to these questions provide information on the following:

- The type of micromobility used including motorcycle, moped, bicycle, scooter, e-moped, e-motorcycle, e-bicycle, and e-scooter.
- The behavior of micromobility users including normal, aggressive, and dangerous behavior.
- The PE (PE-preventive) used including high-visibility clothing, front light, rear light, reflective accessories, mirrors, and bells.
- The PE (PE-protective) used including helmet, gloves, glasses, body armor, chest guard, back protector, elbow pads, knee pads, and foot protector.
- Accidents experienced by users including crashes, near misses, and falls.
- Sustained injuries by users including head, face, chest, back, arms, hands, legs, and foot injuries.
- The severity of sustained injury including minor, moderate, and serious injury.
- The cause of experienced accident such as users are not using accident prevention tools, users are invisible to other road users, and other road users are invisible to the user.
- The performance of PE-preventive such as high-visibility clothing is supportive and useful, while the mirror is not.
- The performance of the PE protective used such as helmets and gloves are effective.

Effectively, micromobility users can respond to this questionnaire and answer questions either before they start their test rides or at the end of their trip.

### 3.2  Data processing and preparation

In the data collection stage, the collected data related to a micromobility user include the user’s response to the web-based questionnaire, the user’s micro-vehicle sensor kit data, and the hazards experienced by the user, such as the number of dangerous overtakes per time unit (output of the micromobility hazard detector). Effectively, the responses of micromobility users to some of the questionnaire questions may require coding or transformation, such as identifying the general behavior of users depending on the behaviors and actions of these users when they use micro-vehicles. However, the readings of environmental sensors, such as temperature and humidity, may be translated into weather condition features, such as good, fair, or poor. Additionally, camera footage can be viewed and manually analyzed to detect and identify the number of near misses (i.e., per time unit) experienced by users during their test rides.

**Figure 2.**
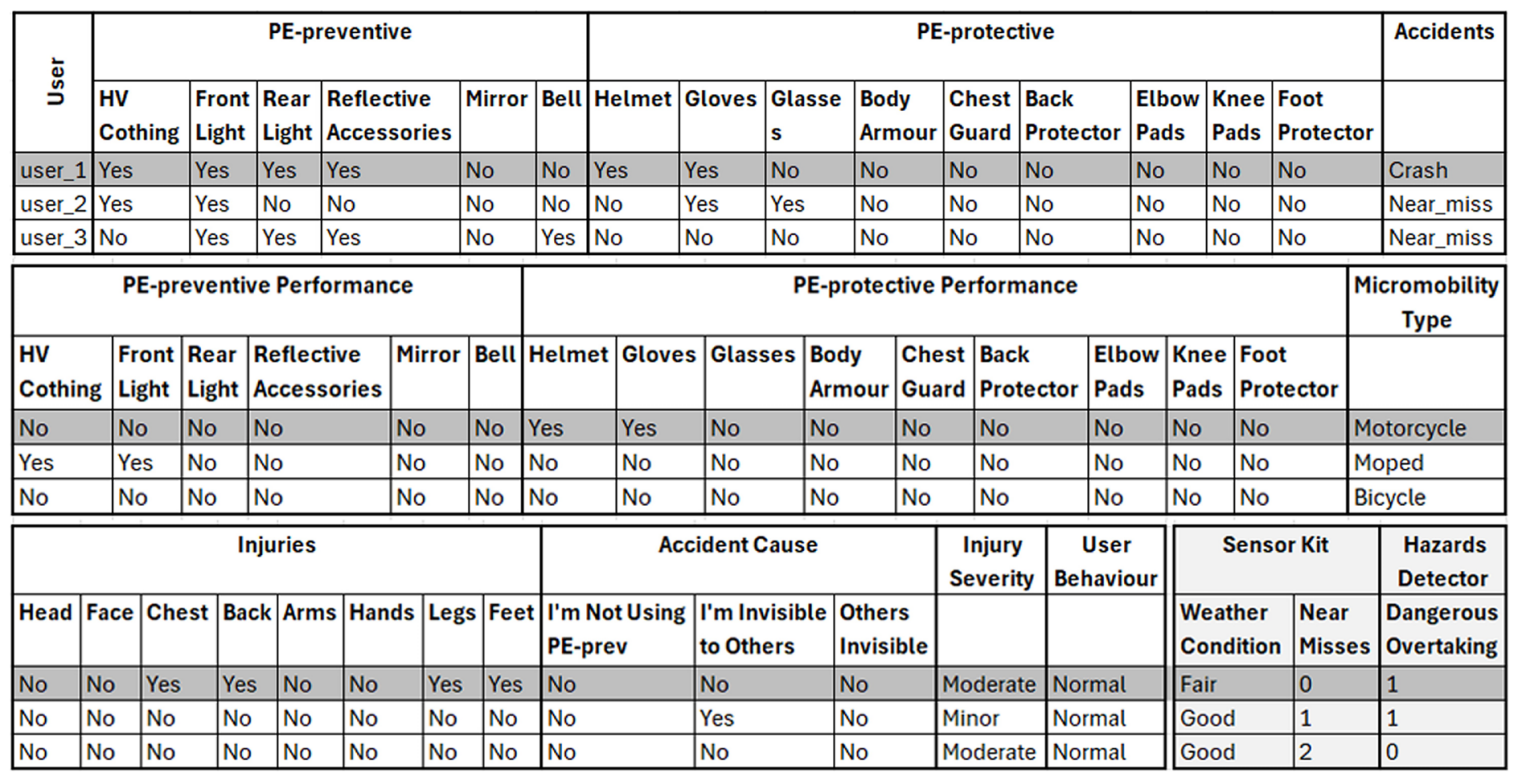
An example of the collected data after processing and preparation.

The data processing and preparation stage starts after all the required data are collected from the different data sources, including the responses of all micromobility users and the related data from the micro-vehicle sensor kit and micromobility hazard detector. All collected data will be linked, organized, and combined in one dataset. Therefore, each row in this dataset includes all the collected data for one micromobility user. Figure 2 represents an example of this dataset and shows that each data entry (i.e., shaded row) includes the answers of one micromobility user to the questionnaire, weather conditions, number of near misses, and number of dangerous overtakes (per time unit).

### 3.3  Data analysis and PE effectiveness evaluation

Evaluating the effectiveness of micromobility PE aims to improve the safety of micromobility and VRUs. This evaluation may enhance users’ awareness of the benefits of using PE and encourage them to wear and use PE constantly while riding micro-vehicles. Therefore, all response data to the questionnaire, micro-vehicle sensor kit data, and micromobility hazard detector data will be analyzed after they have been processed and prepared to assess the efficacy of each PE.

A correlation metric is a statistical measure that can be used to understand the strength and direction of the relationship between two or more variables in data. Therefore, quantifying this relationship is vital for decision-making regarding the effectiveness of different PE. Hence, correlating the metrics related to the surroundings of micro-vehicles with the characteristics and features of micromobility users and the use of PE is crucial in the data analysis process for exploring the relationships among the data variables listed in Figure 2.

**Figure 3.**
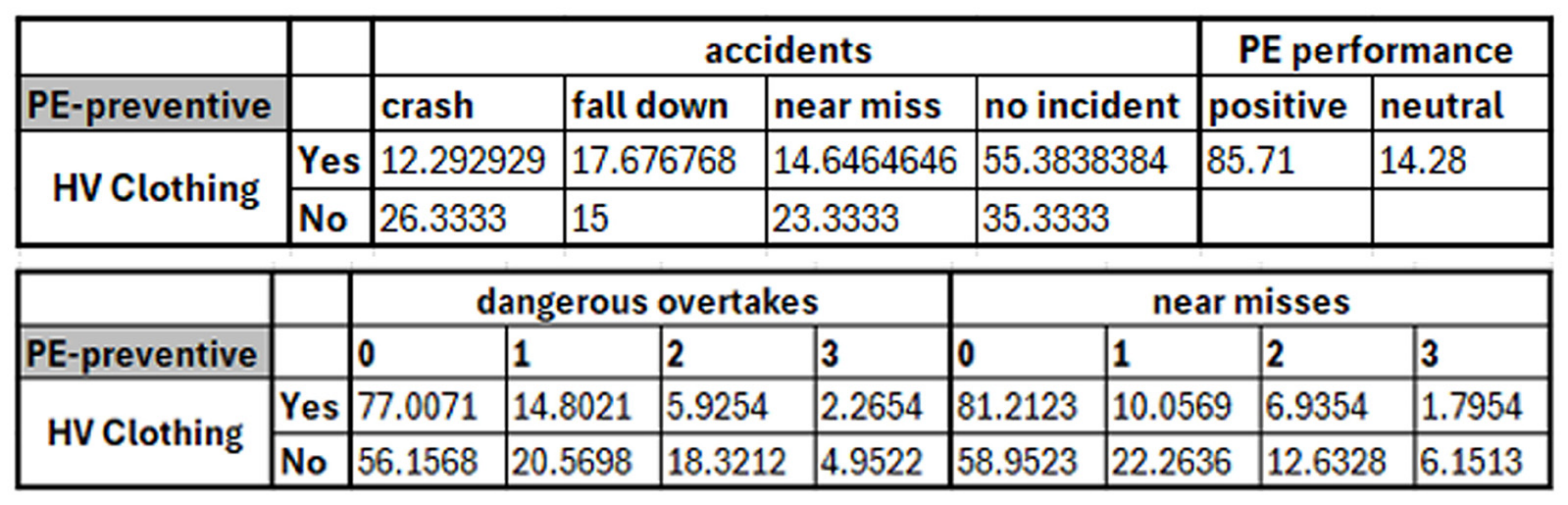
Examples of generated tables to measure the effectiveness of high visibility clothing.

Effectively, the correlations among the collected data variables can be measured to understand the relationships among these variables and how they affect each other. However, some questions in the designed questionnaire may be used to identify the relationships that need to be measured. For instance, the question of “how effective is high-visibility clothing in preventing or reducing accidents?” can be answered by analyzing and quantifying the relationship between PE usage, PE performance, accidents experienced, number of experienced near misses, and dangerous overtakes. In the data analysis and metrics correlation process, the statistics related to each data variable must be calculated and organized in an independent table to estimate the relationships among the relevant variables and draw meaningful conclusions. To answer this question, Figure 3 shows an example of the generated tables containing the statistics of different variables. For instance, the figure shows that only 14.64% of micromobility users who are usually wear high-visibility clothing have experienced near miss incidents, whereas 23.33% of those who don’t usually wear this PE have experienced near miss incidents (historical data – questionnaire response data). Therefore, wearing this PE can reduce the experienced near misses and enhance the safety of micromobility users. Additionally, the remaining relationships between the usage of this PE and other data variables (i.e., PE performance, measured near misses, and dangerous overtakes) can be investigated and discussed in a similar manner.

## 4.  Experiments and Results

The proposed approach for evaluating the effectiveness of micromobility PE requires various types of data to be collected using different techniques. Effectively, the data collection process is key and represents the most challenging aspect of the proposed approach. Because the proposed approach relies on correlation metrics and statistics, implementation of the proposed data collection plan requires a substantial number of micromobility users (i.e., cyclists or e-scooter riders) to be recruited, and each user should be provided with an equipped (with a sensor kit) micro-vehicle. However, the proposed data collection plan has not yet been implemented to collect real input data. Therefore, there are no real data that can be used to evaluate and test the proposed approach for the effectiveness evaluation of micromobility PE.

Simulations and algorithms can be used to create synthetic or artificial data that mirrors real data. Therefore, these synthetic data can be used to replace or expand real-world data. Synthetic data were generated to test the proposed approach and demonstrate the different steps and procedures involved in this approach. Therefore, the performance of the proposed approach can be provisionally evaluated by conducting laboratory experiments and using these synthetic data. However, these synthetic data were created to represent the final format of the collected data before the data analysis and PE effectiveness evaluation step, and after the data processing and preparation step ( Figure 2).

To generate synthetic data, a sample dataset was manually created, and each entry in this dataset represented all the data types expected from all data sources (i.e., questionnaire, micro-vehicle sensor kit, and micromobility hazard detector). Most potential values for the different data variables were considered in this sample dataset, such as types of micromobility, types of user behavior, types of accidents experienced, and sustained injuries. However, the size of this manually generated sample dataset is still limited (i.e., 48 entries) and insufficient to extract significant conclusions. Generally, some techniques can be used to learn from real data and generate synthetic data with high fidelity. One example of these techniques is the Conditional Tabular Generative Adversarial Network (CTGAN), which is a collection of deep-learning-based synthetic data generators for single-table data ( Ince *et al.*, 2024). Therefore, CTGAN was adopted and used to expand the manually generated sample dataset (i.e., a total of 498 entries). As a result, the format of the synthetic data generated is similar to that shown in Figure 2 but includes data for 498 users.

**Figure 4.**
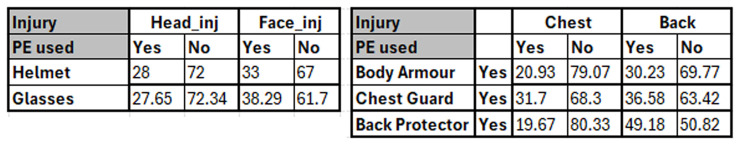
An example of generated tables to measure the effectiveness of different protective equipment.

Having generated the required data in a format similar to that of processed and prepared data, these data are now ready to be analyzed to evaluate the effectiveness of PE. Therefore, the data analysis procedures and techniques described in Section 3.3 can be followed and applied to assess the effectiveness of different micromobility PE. Essentially, the correlations among data variables are measured, and the statistics related to these data variables are calculated and organized in tables to estimate the relationships among these variables and extract the results. Figure 4 shows an example of these tables to quantify the relationship between the use of micromobility PE and the injuries sustained by these micromobility users. For instance, it can be concluded from these tables that micromobility users who are usually wearing helmets can significantly reduce the chance of head and face injury. This is true, at least for all the PE listed in the tables (i.e., glasses, body armor, chest guard, and back protector), in terms of reducing the risks of injury and enhancing safety.

**Figure 5.**
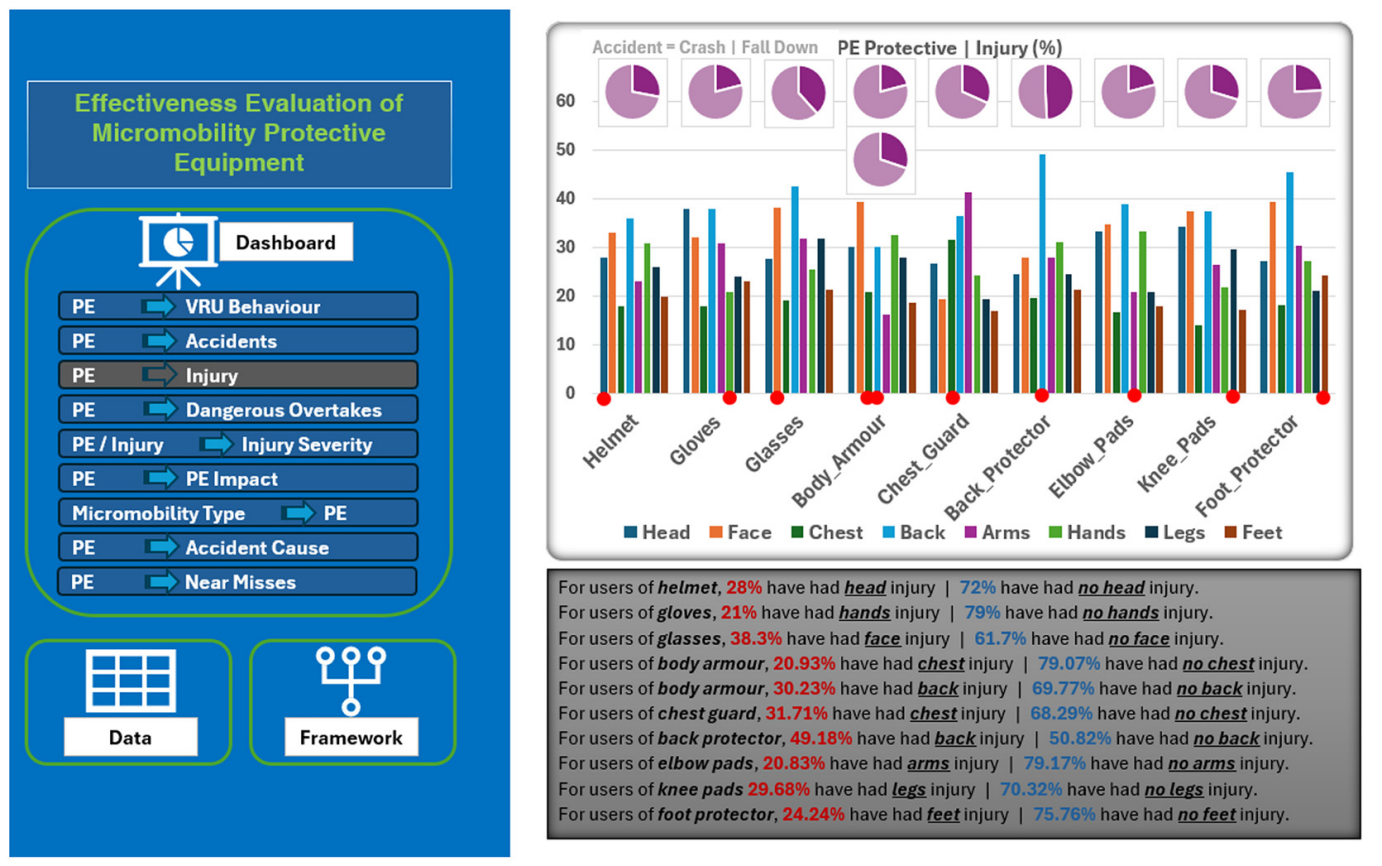
A screenshot for the Microsoft Excel-based dashboard designed to show the effectiveness of protective equipment.

Effectively, a wide range of relationships among the data variables has been identified in a manner similar to what has been explained above. In addition, a static Microsoft Excel-based dashboard was implemented and used as a basic visualization and presentation tool for these relationships ( Figure 5). Therefore, all the generated tables and associated charts were incorporated in this dashboard to make the relationships among the data variables easily understandable. The left side of the dashboard shows the measured relationships between the PE used from one side and the other data variables from the other side. However, the right side of this dashboard shows the selected relationship (in this example, between the used PE and the sustained injury type) in terms of the associated charts and the interpretation of this relationship. This dashboard enables micromobility users (or other stakeholders) to select which relationship to display, understand the meaning of this relationship, and recognize the effectiveness of each PE.

## 5.  Conclusion

The safety of micromobility users, such as cyclists and e-scooter riders, is a key factor in adopting this mode of transportation. Therefore, micromobility users may use or wear various types of PE, including helmets, high-visibility clothing, and lights, to improve their safety. In this paper, we presented a novel approach for evaluating the effectiveness of micromobility PE. Therefore, the aim of our approach is to provide micromobility users and other stakeholders with insights about the benefits and usefulness of PE and whether each PE achieves its objectives. The proposed data-driven approach evaluates the effectiveness of various types of PE, including PE-preventive (i.e., light and reflectors) and PE-protective (i.e., helmets and gloves). It is mainly based on collecting data from micromobility users, micro-vehicle sensor kit, and micromobility hazard detector. Therefore, the collected data were processed and analyzed before measuring the relationships and calculating the correlations among the data variables. Additionally, these relationships among the data variables were presented and visualized to show the effectiveness of various PE. As a next step, we are looking to put the data collection plan into practice; therefore, the real data required for the functionalities of the PE effectiveness evaluation method can be collected. Therefore, the proposed method can be implemented, correlations among data variables can be quantified, and actual results and conclusions can be extracted.

## 6.  Ethics and consent

Ethical approval and consent were not required.

## Data Availability

The data for all the experiments presented in the paper are available via accessible links on the Zenodo platform: Manually created data, and each entry in this dataset represented all the data types expected from all data sources (i.e., questionnaire, micro-vehicle sensor kit, and micromobility hazard detector), https://doi.org/10.5281/zenodo.16794254 ( Almohammad *et al.*, 2025b) Synthetic data generated by using Conditional Tabular Generative Adversarial Network (CTGAN) to expand the manually created data, https://doi.org/10.5281/zenodo.16794356 ( Almohammad *et al.*, 2025c) Data are available under the terms of the Creative Commons Attribution 4.0 International license (CC-BY 4.0).
