# Supplementary material for: A new method for evaluating the effectiveness of micro-mobility protective equipment
Source: Open Res Eur. 2025 Nov 12;5:254. Originally published 2025 Aug 26. [Version 2] doi: 10.12688/openreseurope.20804.2 (PMC12728345; doi:10.12688/openreseurope.20804.2)
Supplement: Peer review history

# Reviewer report: Reviewer response for version 2

**DOI:** 10.21956/openreseurope.23656.r66636

**Published:** 2026-01-05

**Authors:** Azzuhana Roslan

**Affiliations:** 1 Malaysian Institute of Road Safety Research (MIROS), Kajang, Malaysia

The article presents a novel data-driven framework for evaluating the effectiveness of micro mobility protective equipment (PE), integrating three key data sources: a micro-vehicle sensor kit, a micro mobility hazard detector, and a web-based questionnaire.

 The method is designed to overcome limitations of existing studies that rely heavily on retrospective hospital or police data and typically focus on only one category of protective equipment. By combining near real-time sensor information on riding conditions and hazards with self-reported user behaviour and PE usage, the proposed approach enables simultaneous evaluation of both preventive PE (e.g. lights, reflective accessories) and protective PE (e.g. helmets, gloves, body armour). The workflow is logically structured from data collection to processing and preparation, and finally to statistical analysis using phi-coefficient correlation metrics, making the technical description sound and internally consistent.

 The rationale for developing the new method is clearly articulated, highlighting the fragmented nature of previous research and the need for a holistic, context-aware evaluation of PE effectiveness. The paper also provides sufficient operational details to allow replication, including specifications of the sensor kit components, an explanation of the hazard-detection process, a full questionnaire, and open access to the synthetic datasets and manually created seed data used in the proof-of-concept. These elements allow other researchers to reproduce the demonstration stage of the method and to understand how different data streams are integrated and analysed.

 However, although all datasets used in the paper are publicly available, the study relies entirely on synthetic data. As such, reproducibility is only partial and limited to the demonstration environment, and the absence of real-world data restricts the empirical validation of the method. To address this, the authors should include a dedicated real-world validation protocol specifying minimum sample sizes, deployment duration, sensor calibration and data quality procedures, as well as a clear data schema for operational use. In addition, the conclusions, while appropriately cautious, would be strengthened by defining explicit performance benchmarks and decision thresholds (for example, what correlation levels would be considered meaningful in practice) and by providing a worked template showing how real-world results would be interpreted. With these additions, the article would move from a strong proof-of-concept to a fully operational and scientifically robust methodology suitable for wider adoption.

Is the rationale for developing the new method (or application) clearly explained?

Yes

Is the description of the method technically sound?

Yes

Are the conclusions about the method and its performance adequately supported by the findings presented in the article?

Yes

If any results are presented, are all the source data underlying the results available to ensure full reproducibility?

Partly

Are sufficient details provided to allow replication of the method development and its use by others?

Yes

Reviewer Expertise:

Road safety and traffic injury prevention; micromobility and active mobility planning; evaluation of protective equipment and user behaviour; transport safety data analytics; urban mobility systems and policy; application of sensor-based and questionnaire-based methods for transport safety assessment.

I confirm that I have read this submission and believe that I have an appropriate level of expertise to confirm that it is of an acceptable scientific standard, however I have significant reservations, as outlined above.

# Reviewer report: Reviewer response for version 2

**DOI:** 10.21956/openreseurope.23656.r64019

**Published:** 2025-12-23

**Authors:** George Yannis, Virginia Petraki

**Affiliations:** 1 National Technical University of Athens, Athens, Greece; 2 National Technical University of Athens Ship Design Lab, Zografou, Attica, Greece

Thank you for sending the revised manuscript. All of my comments have been fully addressed, and I have no further comments. The manuscript is now suitable for indexing.

Is the rationale for developing the new method (or application) clearly explained?

Partly

Is the description of the method technically sound?

Partly

Are the conclusions about the method and its performance adequately supported by the findings presented in the article?

Yes

If any results are presented, are all the source data underlying the results available to ensure full reproducibility?

Partly

Are sufficient details provided to allow replication of the method development and its use by others?

Partly

Reviewer Expertise:

NA

We confirm that we have read this submission and believe that we have an appropriate level of expertise to confirm that it is of an acceptable scientific standard.

# Reviewer report: Reviewer response for version 2

**DOI:** 10.21956/openreseurope.23656.r64018

**Published:** 2025-11-24

**Authors:** Mariano Gallo

**Affiliations:** 1 Università del Sannio, Benevento, Italy

In my opinion, now the paper can be indexed

Is the rationale for developing the new method (or application) clearly explained?

Yes

Is the description of the method technically sound?

Yes

Are the conclusions about the method and its performance adequately supported by the findings presented in the article?

Partly

If any results are presented, are all the source data underlying the results available to ensure full reproducibility?

Partly

Are sufficient details provided to allow replication of the method development and its use by others?

Yes

Reviewer Expertise:

Transportation engineering

I confirm that I have read this submission and believe that I have an appropriate level of expertise to confirm that it is of an acceptable scientific standard.

# Reviewer report: Reviewer response for version 1

**DOI:** 10.21956/openreseurope.22508.r59752

**Published:** 2025-10-09

**Authors:** George Yannis, Virginia Petraki

**Affiliations:** 1 National Technical University of Athens, Athens, Greece; 2 National Technical University of Athens Ship Design Lab, Zografou, Attica, Greece

**Your Report:**

 This paper is a method article and aims to present a novel approach for evaluating the effectiveness of micromobility protective equipment. The paper is well organized with an interesting objective; however, several key issues should be tackled before the paper is accepted for indexing.

- First, the current dataset is entirely synthetic, built from a manually curated dataset and expanded via CTGAN; no real data have yet been collected. As a methods article, this is acceptable for demonstration, but any empirical claims about PE effectiveness must be removed or recast as illustrative only, with an explicit validation plan for real deployments. Recast all results as a proof-of-concept demonstration, not as empirical findings on PE effectiveness. Avoid language implying real-world effects (e.g., “helmets significantly reduce head/face injury” drawn from synthetic tables).
- Please add a dedicated section that specifies exactly how you will test the effectiveness of the PE metrics once real data is available. The current statement that “a correlation metric can be used…” is too generic; name the specific metrics/models, units, and decision rules. This addition will make the manuscript actionable as a methods article by presenting exactly what metrics to compute, which statistical tests, and how much data is needed (based on literature) to credibly evaluate PE effectiveness.
- Please add a short paragraph that transparently describes how the initial, manually created dataset was built before CTGAN expansion.
- Figure 2 is difficult to read; if a single consolidated table isn’t feasible, keep the three sub-tables but make the linkage explicit (e.g., repeat the user_id).
- Please include in an Appendix the exact questionnaire (full item wording, response options/scales) and cite any validated scales used. This will improve reproducibility and help readers interpret variables and coding choices.
- It is recommended to add a subsection that states exactly what technology you will install on each micro-vehicle, how it will be installed and operated, and for how long.
- Please add a Discussion Section to interpret the contribution in context, relate it to prior work, articulate practical and policy implications, and outline a concrete validation roadmap and future research.

Is the rationale for developing the new method (or application) clearly explained?

Partly

Is the description of the method technically sound?

Partly

Are the conclusions about the method and its performance adequately supported by the findings presented in the article?

Yes

If any results are presented, are all the source data underlying the results available to ensure full reproducibility?

Partly

Are sufficient details provided to allow replication of the method development and its use by others?

Partly

Reviewer Expertise:

NA

We confirm that we have read this submission and believe that we have an appropriate level of expertise to confirm that it is of an acceptable scientific standard, however we have significant reservations, as outlined above.

# Response

**Published:** 2025-11-06

**Authors:** Adel Almohammad

**Affiliations:** School of Architecture and Built Environment, University of Wolverhampton Faculty of Science and Engineering, Wolverhampton, England, UK

Thank you for giving us the opportunity to submit a revised version of our manuscript to Open Research Europe. We appreciate the time and effort that you have dedicated to providing your valuable feedback on our manuscript. We are grateful to the reviewers for their insightful comments on our paper. We have been able to incorporate changes to reflect most of the suggestions provided by the reviewers. We have highlighted the following changes within the manuscript - The results of the study are recasted as a proof-of-concept demonstration (not empirical findings)

 - A section is added to explain the metrics used to evaluate the effectiveness of protective equipment

 - The method used by authors to provide the initial manually created dataset 

 - The tables in Figure 2 are linked by repeating "User" field in all tables

 - The web-based questionnaire designed for data collection is provided

 - A section (Discussion) is added to interpret the paper contribution and outline next research steps

# Reviewer report: Reviewer response for version 1

**DOI:** 10.21956/openreseurope.22508.r59257

**Published:** 2025-09-25

**Authors:** Mariano Gallo

**Affiliations:** 1 Università del Sannio, Benevento, Italy

The paper proposes a new methodology for assessing the effects of protective equipment for micromobility users. The paper is well written and the content is interesting. In the abstract, there is a sentence that, in my opinion, is unrealistic: ‘Micromobility is one of the most important modes of transportation in urban environments...’

 It is more appropriate to say that it is a new and increasingly popular mode of transport, but to say that it is one of the most important is an excess. The idea proposed by the authors is interesting. The most critical point of the paper is the lack of real data, as highlighted by the authors. In practice, it is not possible to be certain that the method will produce accurate results. However, I believe that the paper can be indexed if the authors clarify this aspect in the abstract and show that the equipment to be used is available and the user survey has been designed. In this way, it can be demonstrated that this work is a useful piece of a larger, ongoing but appropriately designed research project.

Is the rationale for developing the new method (or application) clearly explained?

Yes

Is the description of the method technically sound?

Yes

Are the conclusions about the method and its performance adequately supported by the findings presented in the article?

Partly

If any results are presented, are all the source data underlying the results available to ensure full reproducibility?

Partly

Are sufficient details provided to allow replication of the method development and its use by others?

Yes

Reviewer Expertise:

Transportation engineering

I confirm that I have read this submission and believe that I have an appropriate level of expertise to confirm that it is of an acceptable scientific standard, however I have significant reservations, as outlined above.

# Response

**Published:** 2025-11-06

**Authors:** Adel Almohammad

**Affiliations:** School of Architecture and Built Environment, University of Wolverhampton Faculty of Science and Engineering, Wolverhampton, England, UK

Thank you for giving us the opportunity to submit a revised version of our manuscript to Open Research Europe. We appreciate the time and effort that you have dedicated to providing your valuable feedback on our manuscript. We are grateful to the reviewers for their insightful comments on our paper. We have been able to incorporate changes to reflect most of the suggestions provided by the reviewers. We have highlighted the changes within the manuscript.
